# Tyrosine Kinase Inhibitors in Adult Glioblastoma: An (Un)Closed Chapter?

**DOI:** 10.3390/cancers13225799

**Published:** 2021-11-18

**Authors:** Paula Aldaz, Imanol Arozarena

**Affiliations:** 1 Cancer Signaling Unit, Navarrabiomed, Hospital Universitario de Navarra (HUN), Universidad Pública de Navarra (UPNA), 31008 Pamplona, Spain; 2 Health Research Institute of Navarre (IdiSNA), 31008 Pamplona, Spain

**Keywords:** glioblastoma, receptor tyrosine kinase, tyrosine kinase inhibitors, targeted therapy

## Abstract

**Simple Summary:**

Glioblastoma multiforme (GBM) is the most common type of malignant brain tumor. GBM patients face a dire future, as they rarely survive longer than 15 months after diagnosis. Typically, patients undergo surgery to remove the tumor followed by combined radiotherapy and chemotherapy. However, these therapies usually extend survival only for several months, since tumors invariably regrow, which is called recurrence. Targeted therapies against specific genes that control GBM tumor-growth have been tested in clinical trials for years without success. In this article, we describe the main scientific findings leading to the testing of these targeted therapies in GBM, and discuss the potential causes behind the failure of these clinical trials. We highlight the importance of performing molecular analyses in tumors to determine the presence of those genes controlling GBM growth, before administering drugs specifically blocking their activity. In doing so clinicians could identify patients that could potentially benefit the most. Furthermore, we discuss the reasons to test these drugs in newly diagnosed patients rather than in patients under recurrence. In summary, the aim of this review is to propose alternative approaches for the design of clinical trials testing targeted therapies in GBM patients based on available scientific evidence.

**Abstract:**

Glioblastoma (GBM) is the most common and lethal form of malignant brain tumor. GBM patients normally undergo surgery plus adjuvant radiotherapy followed by chemotherapy. Numerous studies into the molecular events driving GBM highlight the central role played by the Epidermal Growth Factor Receptor (EGFR), as well as the Platelet-derived Growth Factor Receptors PDGFRA and PDGFRB in tumor initiation and progression. Despite strong preclinical evidence for the therapeutic potential of tyrosine kinase inhibitors (TKIs) that target EGFR, PDGFRs, and other tyrosine kinases, clinical trials performed during the last 20 years have not led to the desired therapeutic breakthrough for GBM patients. While clinical trials are still ongoing, in the medical community there is the perception of TKIs as a lost opportunity in the fight against GBM. In this article, we review the scientific rationale for the use of TKIs targeting glioma drivers. We critically analyze the potential causes for the failure of TKIs in the treatment of GBM, and we propose alternative approaches to the clinical evaluation of TKIs in GBM patients.

## 1. Introduction

Glioblastoma multiforme (GBM) is the most common and malignant adult primary tumor, with an annual incidence rate of 3.23 per 100,000, accounting for almost 50% of all malignant brain tumors [1]. There are 20,000 new cases diagnosed in the United States of America every year; nearly half of the patients are over 65, although GBM can occur at any age [2]. GBM remains among the cancers with poorest prognosis, with a 15-month median overall survival (OS) after diagnosis and a 5-year survival rate below 7% [1]. Conventional management of GBM consists of surgical removal of the tumor, followed by combined radiotherapy and chemotherapy, and usually six monthly cycles of adjuvant temozolomide (TMZ) [3]. However, adjuvant therapy protocols have low success rates, extending survival for just three months, as patients invariably relapse [4]. Recurrence rates reach around 90%, and, in these cases, GBM recurs with a poorer prognosis (i.e., median PFS of 1.5–6 months and median OS of 2–9 months) [5,6]. Recurrences are mostly local, within 2 cm of the initial tumor margin, usually are not accessible to surgery [5], and are less sensitive to therapy than the original tumor; indeed there is no standard treatment for recurrent GBM. 

The World Health Organization (WHO) classifies gliomas into four degrees of malignancy, based on histopathological and clinical criteria [7]. GBMs are categorized as grade IV gliomas, characterized by high mitotic activity, microvascular proliferation, necrosis, resistance to apoptosis, invasion to adjacent brain tissue, and accumulation of genomic aberrations [7,8,9]. From a clinical point of view, GBMs have been historically differentiated into two groups: primary and secondary. 

Primary or de novo cases occur without clinical evidence of a lower-grade glioma (LGG), and form the majority (90%) of cases, while secondary cases derive from the progression of LGGs [10]. Recently, however, the WHO classification has been updated based on advanced molecular analyses in order to improve the precision of the diagnosis of tumors. This new classification takes into account both the molecular and histological characteristics: it integrates the genotypic and phenotypic parameters and classifies GBMs based on their isocitrate dehydrogenase (*IDH*) mutation status as *IDH*-wild type, *IDH*-mutant, or as GBM with an inconclusive or unavailable *IDH* mutation status, such as GBM NOS (not otherwise specified) [11]. *IDH*-wild type GBM displays mutations in the telomerase reverse transcriptase (*TERT*) promoter (72%), in TP53 (27%) and in the phosphatase and tensin homolog (*PTEN*) (24%), as well as amplifications (35%) of the epidermal growth factor receptor (*EGFR*) gene. *IDH*-mutant GBM also harbors mutations in *TP53* (81%), but is characterized by a high degree (71%) in mutations in *ATRX* [11].

Over time, further molecular alterations typical for GBM have been identified and high-throughput molecular analyses have helped to improve a molecular classification of these tumors.

## 2. Molecular Classification of GBM

In the last few decades, ambitious molecular studies have revealed the remarkable degree of inter-tumor heterogeneity present in GBM [12,13,14]. Results from The Cancer Genome Atlas (TCGA) program revealed that the most frequent alterations in GBM predominantly affect three cell-signaling pathways: the p53 pathway, the retinoblastoma (Rb) pathway, and the receptor tyrosine kinase (RTK) signaling pathways [13]. Indeed, copy number alterations of RTKs, such as the *EGFR* or platelet-derived growth factor receptor α (*PDGFRA*), in GBM had been reported already more than 30 years ago [15,16]. EGFR expression is common in GBM, and tumors characterized by overexpression of the EGFR can predict poor patient survival [17]. Following the detection of EGFR amplifications, gene signatures associated with EGFR overexpression and other GBM clinical features have been defined [17,18,19]. These signatures also helped to identify molecular subclasses such as proneural, mesenchymal, and proliferative [20]. In the latter study, EGFR amplifications were predominantly found in tumors of either proliferative or mesenchymal subclasses.

Further characterization by the TCGA led to the definition of four molecular GBM subtypes—mesenchymal, classical, neural, and proneural—taking into account genetic and epigenetic alterations, response to treatments, and prognosis [21]. Thereby, the mesenchymal subtype is linked to mutation or deletion of the two well-known tumor suppressor genes neurofibromin 1 (*NF1*) and *PTEN*. *NF1* encodes a Ras-GTPase activating protein that blocks RAS signaling, while the product of PTEN suppresses the activation of the PI3K pathway downstream of RAS [22]. The classical subtype is associated with amplification and mutation of *EGFR*, deletion of the cyclin-dependent kinase inhibitors *CDKN2A* and *2B*, and gain of chromosome 7 concomitant with loss of chromosome 10. The neural subtype also displays *EGFR* amplification and *PTEN* deletions, and the proneural subtype is associated with *PDGFRA* gene mutation and/or amplification, as well as *TP53* and *IDH1* mutations.

Importantly, distinct cells in the same tumor often recapitulate programs from distinct subtypes, and these subtypes can co-exist in different regions of the same tumor, leading to intratumor heterogeneity [14,23,24]. Nevertheless, subtypes can change during tumor development and also through therapy, whereby disease progression and therapy resistance is predominantly linked to the mesenchymal phenotype [20,25].

Recently, an integrative unified model of cellular states, genetics, and plasticity has been described [26]. Single-cell lineage tracing and expression analysis described that malignant cells in GBM exist in four main cellular states that are related to distinct neural cell types. The individual cellular states exhibit plasticity, and their establishment is influenced by signals from the tumor microenvironment. The distinct states have been described as neural progenitor-like (NPC-like), oligodendrocyte-progenitor-like (OPC-like), astrocyte-like (AC-like) and, finally, mesenchymal-like (MES-like) states. The relative frequency of cells in each state is influenced by copy number amplifications of the *CDK4*, *EGFR* and *PDGFRA* loci, and by mutations in the *NF1* locus [26]. For instance, TCGA tumors with high-level genetic amplification of EGFR are associated with higher AC-like bulk scores. High-level amplifications of PDGFRA and CDK4 are associated with OPC-like and NPC regulators, and NF1 alterations were correlated with MES-high tumors [26].

Apart from gene amplification, GBM presents EGFR deletions and point mutations. There are several variants of EGFR, defined by the deletion of different exons: EGFRvI (deletion of exons 1–13) [27]; EGFRvII (exons 14 and 15 deletion) [28]; EGFRvIII (exon 2 to 7); EGFRvIV (exons 25–27); and EGFRvV (exons 25–28) [29]. The protein product of the EGFRvIII variant lacks a sequence of 267 amino acids in the extracellular ligand-binding domain, leading to a constitutive activation of the EGFR pathway. This variant is the most common, and can be found in 20–50% of GBMs that carry EGFR amplification [13]. Furthermore, EGFR point mutations have been identified in almost 25% of GBM samples. Such mutations include the substitution of arginine by lysine at position 108 (R108K), the substitution of an alanine at residue 289 by valine, aspartic acid or threonine (A289V/D/T), and the substitution of glycine by aspartic acid at position 598 (G598D). These mutations lead to a constitutively active kinase activity [30].

Regarding PDGFRA, it has been shown to be altered by overexpression, amplification, mutation, or rearrangements. PDGFRA gene rearrangements, such as fusion between kinase insert domain receptor (KDR) (VEGFRII) and PDGFRA gene, have been found in PDGFRA-amplified GBM. Intragenic deletion rearrangements such as PDGFRA^Δ8, 9^, which is formed by an in-frame deletion of 243 bp in exons 8 and 9 of the extracellular domain, were observed in 40% of the GBM with PDGFRA amplification [31]. Different studies have also identified PDGFRA point mutations localized in different domains of the receptor, mainly in the Ig-like extracellular domain, such as C235Y and W349C, potentially disrupting ligand interaction [21]. More mutations have been reported in the extracellular domain, such as Y288H and the activating mutations Y288C and P345S. In addition, activating mutations like V561D in the juxtamembrane and D842V in kinase domain have been encountered, In addition to R1037K mutation, which is found in the intracellular kinase domain, and s frequent in 1 of 316 samples [32]. Some of those mutations, such as V536E, which can be found in the transmembrane domain, have been functionally characterized, and have reported a gain of function by stimulating cell growth and signaling via ERK and STAT5 in the absence of ligand [33].

Although currently not considered for GBM tumor subtyping, in 50% of cases with EGFR amplifications, tumors also carry a truncated form of EGFR that possesses constitutive kinase activity, which is named EGFRvIII, or otherwise EGFR type III, de2–7, ΔEGFR) [34]. Increased EGFRvIII signaling has been correlated with glioma progression and poor prognosis. Finally the RTK MET (Mesenchymal–Epithelial Transition factor) is also amplified in a significant proportion of gliomas, and appears to be co-amplified with EGFRvIII [35]. Apart from MET amplification and overexpression, a mutant form of MET, METex14 is present in 14% of secondary GBM and is characterized by the deletion of exon 14 in the intracellular domain, which harbors the binding site for Cbl. As a consequence, METex14 shows constitutive activation and decreased protein degradation [36].

Altered expression of fibroblast growth factors receptors (FGFRs) has also been described in GBM. For instance, FGFR1 and FGFR2 gene amplification, abnormal activation, or single nucleotide polymorphisms (SNPs) [37]. In addition, oncogenic fusions of FGFR3 and FGFR1 to the transforming acidic coiled-coil (TACC) proteins, which generate oncogenic forms, have been reported [38]. In fact, FGFR3-TACC3 fusion gene is present in 3% of GBMs [38]. Recently, a comparative integrated analysis found four new clinically actionable alterations in FGFR2 and FGFR3 that promote an aggressive phenotype [39].

## 3. RTK as Drivers in GBM

### 3.1. RTK Signalling

EGFR, also known as ErbB1/HER1, is one of four members of the EGFR/ErbB family in humans. The other members are ErbB2/HER2, ErbB3/HER3, which have an impaired kinase domain, and ErbB4/HER4. The receptors can form homo- and hetero dimers, depending on the ligands they interact with. Ligand binding induces dimerization, which results in a conformational change, and kinase domain activation followed by auto/trans-phosphorylation events at specific tyrosine residues that fully activate the individual receptors [40]. Ligands that induce EGFR homodimers are EGF itself, transforming growth factor α (TGF-α), amphiregulin (ARG), and epigen (EGN) [40]. After ligand binding, the fully activated EGFR can bind, and phosphorylate a wide range of effectors, including kinases of the SRC family, PLC gamma, the RAC guanine-nucleotide exchange factor VAV, transcription factors such as STAT5, and, most importantly, adaptor proteins that trigger the activation of the RAS GTPases and subsequent stimulation of the RAF/MAPK and PI3K-AKT pathways. EGFRvIII, the truncated form of EGFR, has a deletion of exons 2–7, affecting the extracellular domain. As a consequence, EGFRvIII cannot bind EGF, but shows constitutive activity due to decreased receptor internalization and protein degradation [41]. 

Receptor dimerization also plays a role in PDGFR signaling, whereby PDGFRa and PDGFRb can homo- or heterodimerize. These dimers are activated by binding of platelet-derived growth factor a, b, c, and d (PDGFA, B, C or D), which requires prior dimerization of the ligands [42]. Specifically, PDGFAA, BB, CC and AB ligand dimers activate PDGFRAA homodimers, while the PDGFRAB heterodimer is mostly activated PDGFBB, PDGFAB dimers, and PDGFRBB dimers are activated by PDGFBB and PDGFDD [42]. The binding of PDGFs and activation of PDGFRA/B can occur in a exocrine, paracrine, and autocrine manner [43]. Similarly to the EGFR, ligand-bound PDGFRs auto/trans-phosphorylate specific tyrosine residues, which leads to the binding and activation of many of the abovementioned EGFR effectors, including the RAF/MAK and PI3K/AKT pathways. The RTK MET is a receptor for the hepatocyte growth factor (HGF), and it is synthesized as a single-chain precursor that is cleaved by furin yielding an N-terminal α-chain and a C-terminal β-chain [44]. Active, cleaved HGF binds the alpha and the beta chain to induce receptor dimerization and subsequent activation [45]. Activated, autophoshorylated MET recruits and activates downstream effectors that include SRC kinases and, via RAS, the RAF/MAPK and PI3K/AKT pathways [46].

The duration and intensity of RTK activation is crucial for the establishment of distinct transcriptional programs that impact on cell proliferation, differentiation, survival, migration, invasion etc. Furthermore, such responses are also dependent on cell type specific traits and the context of the tissue in which RTK activation and signaling takes place [47].

Increased knowledge of the structure, activating mechanisms of RTKs and downstream signaling modules have substantially improved our understanding of the cellular machinery that mediates gliomagenesis and maintains the malignant phenotype of transformed glia.

### 3.2. Pre-Clinical Evidence of RTK Involvement in GBM

Preclinical studies on RTK signaling in brain tumors involved established glioma cell lines, xenograft tumors (cell line-based and patient derived) and genetically engineered mouse models (GEMM). Over the years, these models have led to a better understanding of the role of EGFR, PDGFRs, and other RTKs in gliomagenesis as well as glioma progression, and have been crucial for testing new, potentially active therapeutic agents.

The use of GEMMs has demonstrated that deregulated PDGFR or EGFR signaling in an adequate genetic background promotes gliomagenesis [48,49,50]. In order to understand EGFR signaling in GBM and to better predict the efficacy of targeted therapeutics, a variety of preclinical models of GBM based on overexpression of EGFR and EGFRvIII have been developed [51]. The majority of glioma mouse models have employed cre/LoxP technology to create genetic alterations. A modification of this technology incorporates a Cre-mediated multifluorescent protein expressing system, which allows for the dissection of developmental processes of gliomagenesis and detecting morphologically heterogeneous tumor populations in gliomas [52]. Alternative systems to generate targeted mutations in a tissue-specific manner are the RCAS (replication-competent ASLV long terminal repeat with a splice acceptor) [53] and the Sleeping Beauty (SB) transposon/transposase system [54]. Studies using these systems showed that expression of EGFR in adult brain tissues is not a transforming event, but that loss of p16lnk4a, p19Arf, and PTEN cooperates with EGFR in gliomagenesis. They also showed that EGFR signals through its canonical pathways, whereas tumors arising from expression of mutant EGFRvIII do not use these same pathways [51]. A murine PDGFB-driven glioblastoma model based on the RCAS/Tva system [49] has been used to study the effects of corticosteroids within the tumor microenvironment and their negative impact on radiotherapy [55]. A PDGFRα-driven mouse model based on autocrine receptor stimulation revealed that the tubulin-binding protein Stathmin1 is a PDGFRα phospho-regulated target, whose misregulation confers sensitivity to vinblastine (VB) cytotoxicity [56]. In addition, plenty of studies have been made in order to study the role of PDGF ligands in gliomagenesis [57]. For instance, transgenic mice expressing PDGFB on a Tp53 null background develop brain tumors resembling human GBMs [58]. Also injection of RCAS-PDFGA in Pten of Cdkn2a null mice [59]. For instance an animal model of ATRX-deficient GBM was created to show that loss of ATRX accelerated tumor growth rate and reduced median survival [60].

Early genetic approaches utilizing RNA interference suggested that EGFR depletion from glioma cells could induce a partial cell cycle arrest in G2M [61], while later studies showed that treatment with EGFR specific siRNAs had no inhibitory effect on cell proliferation, migration, and activation status of EGFR-coupled signaling cascades [62]. Moreover, sole pharmacological inhibition of EGFR by the tyrosine kinase inhibitor erlotinib displayed no activity in 2D clonogenic survival assays, nor in 3D GBM spheroids [63]. These reports indicate that specific down-regulation or inhibition of EGFR is not sufficient as a single agent therapeutic approach.

However, combining the downregulation of EGFR by siRNA with the up-regulation of PTEN expression in PTEN-deficient U251 cells resulted in cell cycle arrest, suppression of proliferation, reduction in invasion and promotion of apoptosis, and growth reduction of U251 subcutaneous xenografts [64]. Anti-proliferative effects were also seen when cells were treated with the EGFR inhibitor AG1478 in combination with the GSI-X Notch signaling inhibitor [65]. Moreover, a combination of the EGFR inhibitor afatinib with temozolomide significantly decreased xenograft growth and progression of intracranially injected U87EGFRvIII GBM xenografts [66]. Combining an anti-EGFRvIII monoclonal antibody with rapamycin resulted in an inhibition in the growth of U87-EGFRvIII and U251- EGFRvIII cells when injected subcutaneously into nude mice [67]. Furthermore, D2C7-IT, a novel immunotoxin targeting wild-type EGFR as well as EGFRvIII proteins, when combined with checkpoint inhibitors, improved survival in intracranial glioma models [68]. 

Targeting the PDGFRA with a neutralizing human monoclonal antibody inhibits the growth of tumor xenografts [69], but, notably, there is more preclinical evidence linking PDGFR-β to glioma proliferation and survival than PDGFRA. A PDGFR-β-specific shRNA can reduce viability and enhance GBM radiosensitivity [70], and silencing PDGFRB by RNAi was shown to enhance the radiosensitivity of C6 glioma cells in vivo [70]. The PDGFRB inhibitor AG1433 induces cytotoxicity in high grade glioma cell lines [71]. The combination of the PDGFRB inhibitor JNJ-10198409 and the IGF-1R inhibitor PPP/CAS 477-47-4 reduced Akt and Erk1/2 phosphorylation, and diminished cell proliferation, through a G2/M blockade of the cell cycle [72]. In addition, the use of anti-PDGF antibodies also resulted in a reduction of cell viability and induction of autophagy in glioma cells [73].

## 4. Factors Limiting the Effectiveness of TKIs in GBM 

RTK inhibitors of the family of TKIs can be grouped into reversible and irreversible inhibitors. Irreversible TKIs bind their target via covalent bounds, whereas reversible inhibitors are based on non-covalent binding. The latter are subdivided between ATP-competitive inhibitors (Type I) that occupy the ATP-binding pocket, and molecules that bind the inactive form of the RTK adjacently to the ATP pocket (Type II). The ATP-binding domain is structurally well conserved among certain RTKs, which limits the binding specificity of these drugs [74]. Most covalent/irreversible TKI tested in GBMs target EGFR (afatinib, neratininb, osimertinib). As evidenced in Table 1, apart from EGFR inhibitors, the vast majority of drugs trialed so far targeting PDGFR signaling in GBM also block several other RTKs. Indeed, some of them, such as ponatinib, vandetanib, dasatinib, and cabozantinib, can affect the activity of more than eight different kinases [75,76] (Table 1). As a consequence of this lack of specificity, the inhibition of driver kinases is diluted and the probability of off target effects leading to the activation of compensation mechanisms is increased. Furthermore such unspecificity can lead to increased systemic toxicity/adverse effects that limit the treatment duration and, therefore, efficacy [77,78].

### 4.1. Blood Brain Barrier (BBB) and Drug Accumulation

The blood–brain barrier (BBB) is a very selective membrane that limits the entry of drugs, biomolecules, and cells to the central nervous system (CNS). The BBB is bordered by the basal lamina, a glycoprotein-rich extracellular matrix (ECM), also formed of endothelial cells, pericytes, and astrocytes [79]. There are a variety of mechanisms for substances to cross the BBB, such as transmembrane diffusion and active transport among others [80]. Passive diffusion of small molecules through the BBB depends on their lipophilicity; small hydrophobic molecules diffuse transcellularly, whereas small hydrophilic compounds can enter the brain via the paracellular route [79]. Active transport across the BBB endothelium is regulated by ATP-binding cassette transporters (ABC transporters) located within vessel walls. ABC transporters regulate efflux from the endothelium into the luminal compartment. However, these ABC transporters are often responsible of decreasing the uptake rate of potential drugs crossing de BBB, since most anti-neoplastic low molecular weight drugs are substrates for ABC proteins [79]. The most common ABC transporters are P-glycoprotein (P-gp; also known as ABCB1), and breast cancer resistance protein (BCRP; also known as ABCG2) [79]. For instance, brain accumulation of TKIs such as regorafenib, gefitinib, and tivozanib is restricted by P-gp and BCRP [81,82,83]. Some other studies showed that oral administration of Imatinib resulted only in a marginal flux across the blood-brain barrier [84,85]. There are some other drugs, such as axitinib and tesevatinib, that were detected in the brain of the animals, and were able to permeabilize an in vitro BBB model, which strongly suggests that it could efficiently reach human brain tumors [86,87]. Sunitinib, dasatinib, and sorafenib are drugs capable of entering the brain [88,89,90]. Studies assessing the pharmacokinetic of the multikinase inhibitor ponatinib demonstrated that P-gp and BCRP restricted ponatinib brain accumulation [91,92], although a comparative analysis showed that ponatinib has better BBB penetration, and achieves higher brain plasma concentrations than dasatinib [93].

Nevertheless, there is evidence suggesting that the BBB is by no means intact, and that therefore the failure of TKI in GBM cannot be entirely blamed on the BBB blocking drug access to the brain. As shown in Table 2 and Table 3, most clinical trials with TKIs have traditionally recruited patients under recurrence. These patients have undergone surgery and radiotherapy prior to chemotherapy, with both treatments debilitating BBB integrity [94]. Furthermore, tumor-induced/associated neovasculature (the main characteristic of GBM) is leaky and more disorganized than the normal/physiological BBB. However, there is ample evidence that, generally speaking, TKIs do not reach high-enough intra-tumoral therapeutic concentrations. In this regard, the formulation of drugs and the incorporation of nanocarriers and other drug delivery systems might provide renewed hope to chemotherapy in brain tumors. Similarly, local administration of drugs directly into the tumor resection cavity is another strategy being considered in order to bypass the restrictions imposed by the BBB [94,95,96].

### 4.2. Patient Selection

At present, the inclusion criteria used in clinical trials testing TKIs against GBM RTKs are based on the patients status [97]. Most trials consider diverse parameters, such as number of leucocytes and thrombocytes; absence of intracerebral inflammation; adequate hepatic, renal and bone marrow function; and previous treatment received (e.g., surgery undergone), among other factors. The vast majority of trials using small molecules inhibitors targeting RTKs in GBM have not taken into account tumor subtype. In other words, the expression of either EGFR or PDGFRs, drivers of glioma progression, is rarely included when assessing the efficacy of a drug. Only around 15% of all those clinical trials have taken into account expression of RTKs, and 9 out of 10 of them are focused on recurrent GBM rather than newly diagnosed patients [97]. For instance, in an open-label trial of imatinib mesylate with patients with unresectable, recurrent GBM expressing PDGFR (NCT00171938, 2004), immunohistochemical documentation of expression of PDGFR was required for inclusion [97]. In 2012, another multicenter study (NCT01520870) assessed the efficacy and safety of the multi-kinase dacomitinib in patients with recurrent GBM with EGFR gene amplification and/or EGFRvIII mutation, which was determined by in situ hybridization fluorescent (FISH) and/or PCR respectively in the primary tumor [97]. On the other hand, and as the authors acknowledge, no proof of the stability of EGFR amplification in recurrent tumors was provided [97]. More recently, a proof of concept trial investigating crenolanib monotherapy (NCT02626364) in patients with recurrent/refractory GBM included patients with PDGFRA gene amplification, as determined by FISH, at the time of diagnosis or time of recurrence [97]. In 2020, the trial NCT04424966 enrolled participants with recurrent high-grade glioma with FGFR1 K656E or FGFR3 K650E mutations or FGFR3-TACC3 translocation (demonstrated by NGS sequencing, IHC and/or RT- PCR) for the clinical assessment of infigratinib [97].

Historically the majority of clinical trials have focused on recurrent patients compared to newly diagnosed patients (Table 4). However, the proportion of ongoing trials focused on newly diagnosed GBM patients seems to be increasing (Table 4). Notably, less than 25% of the ongoing trials incorporated RTK target expression as inclusion criteria. The NCT03231501 trial started in 2018 to evaluate the EGFR inhibitor epitinib in newly diagnosed patients with EGFR gene amplification [97]. Nevertheless, it is still fairly obvious that the molecular landscape of GBM patients has rarely been used as inclusion criteria.

Certainly, patient stratification based on target/RTK expression is relevant in newly diagnosed patients. These patients are normally subjected to surgery for tumor removal prior to conventional adjuvant therapy. In principle, target expression studies could be carried out, either prospectively or retrospectively. This approach could provide useful insight into the efficacy of TKIs and its correlation with target expression. Unfortunately, such an approach is more difficult to realize in clinical trials recruiting patients with recurrent GBM. These patients are less likely to undergo surgery, and it has become more evident that the genetic landscape of recurrent tumors does not correlate with that of the primary tumor of origin [98,99]. Indeed the mutagenic characteristic of the DNA alkylating agent temozolomide can drive the evolution of recurrent GBM [100]. Similarly, ionizing radiation can change the mutational profile of primary gliomas via the induction of double-strand breaks. Analysis of post-radiation occurring high-grade astrocytomas showed that, compared to spontaneous high-grade gliomas, radiated tumors had an increased prevalence of genomic aneuploidy. This was accompanied by a significant increase in the frequency of PDGFRA, MET, BRAF, and RRAS2 amplifications [101]. Thus there is ample evidence of the potential of current adjuvant therapies to change the genetic landscape of first diagnosed tumor compared to recurrent tumors [102]. Consequently, analysis of primary tumors cannot guide the trial of TKIs on recurrent GBM.

There is clearly a need to develop clinical trials where TKIs are tested as post-surgical adjuvant therapy, whether in newly diagnosed patients or in recurrence. That said, there is lack of information regarding the response of newly diagnosed patients to TKIs. This subset of patients can be easily stratified upon for their tumor molecular profile. Furthermore, newly diagnosed patients are more likely to present a better fitness profile that patients under recurrence [103,104,105].

### 4.3. Tumor Heterogeneity

As mentioned above, distinct cells in the same tumor frequently represent different subtypes, which can co-exist within the tumor, leading to intratumor heterogeneity. GBM tumor subtyping is based on gene amplification of actionable key glioma drivers (50% EGFR amplification in classical or 10–15% PDGFRA amplification in proneural subtypes). However, intratumor heterogeneity leads to an overlap in the expression of these characteristic markers, leading to a mosaic pattern of receptor expression [23]. For instance, mRNA and protein expression studies have shown that high expression and activation of these ‘marker’ RTKs is more widespread than indicated by copy number analyses. Indeed, the PDGFRA protein has been detected in between 25–75% of GBM tumors [106,107], and PDGFRB protein (able to activate similar pathways than PDGFRA via dimerization) may be expressed in up to 60% of cases [106]. Moreover, the ligand PDGFA, which can activate PDGFRA as well as PDGFRB is expressed in up to 80% of tumors [104]. Finally, PDGFR expression is not only dependent on the genetic traits of tumor cells, but can also be regulated by surrounding stromal cell populations such as microglial cells [108].

Using mass spectrometry, Schaff et al. were able to detect and quantify EGFR protein in 48 out of 51 GBM samples, including 22 cases with no EGFR amplification [109]. Verhaak et al. showed that in many tumors of the proneural type EGFR was co-expressed with PDGFRA and vice versa, ‘classical’ tumor types are often positive for PDGFRA. Szerlip et al. described the co-amplification and activation of different RTKs; indeed 43% of GBM with PDGFRA amplification displayed co-amplification of EGFR or MET [110].

This intratumor heterogeneity in RTK expression might reflect what has recently been proposed, namely that GBM tumors represent a dynamic system based on cellular states [26], which adds even more complexity.

Apart from the heterogeneity found amongst individual tumor cells, one must also consider that the tumor cells interact with, and thus shape the tumor microenvironment. This microenvironment comprises astrocytes, neurons, pericytes, endothelial cells, and fibroblasts, as well as immune cells, including macrophages and microglia [111]. Macrophages and the microglia appear to be enriched in tumors with NF1 deficiency, but how this is linked to the expression or activation of particular RTKs is not known [14].

Cells of the tumor microenvironment can be compromised and exploited by the tumor cells. These cells establish a particular extracellular matrix environment and secrete an array of cytokines and growth factors, such as EGF, PDGF, and VEGF, that impact on tissue remodeling and angiogenesis [112], but can potentially also activate RTKs in glioma cells, as it has been established, for instance, in melanoma [113]. Indeed, microglia can stimulate the invasion of glioma cells, and this is partially dependent on EGFR activation [114]. Endothelial cells have been shown to support the propagation of brain tumor stem cells [115], which pose a source for therapy resistance. Importantly however, TKIs such as sunitinib and ponatinib are able to suppress the self-renewing capacity of glioma stem cells [106]. Overall, while the impact of microenvironment on the efficacy of immunotherapies is currently studied, not much is known yet about how it will interfere with the action of TKIs. Nevertheless, it is conceivable that intratumor heterogeneity allows unresponsive tumor cells to escape drug treatment, and that a heterogeneous tumor microenvironment could support drug treated cells, which together poses a crucial challenge to TKI-based therapies in GBM.

### 4.4. Mechanisms of Resistance to TKIs

There are several mechanisms described by which tumor cells withstand RTK inhibition. These involve mutations and target gene amplification, as well as autocrine re-activation of the receptor and mutations in down-stream signaling components.

Due to its high frequency of mutation and overexpression in GBM, EGFR targeting TKIs are widely used in pre-clinical and clinical studies, and have so far provided some inside into resistance mechanisms. A common phenomenon in acquired resistance to EGFRi is the appearance of inhibitor resistant mutations such as the T790M mutation, which hinders first generation ATP-competitive inhibitors to bind the kinase domain [116]. Third-generation irreversible inhibitors such as osimertinib, which is currently being trialed in GBM (Table 1), are thought to overcome such a challenge [117]. However, targeting EGFR in glioblastoma is particularly challenged by the amplification of extrachromosomal DNA, which contributes to significant variability in the expression of EGFRvIII [118]. Epigenetic down-regulation of EGFRvIII expression as response to drug treatment has been demonstrated with erlotinib in pre-clinical GBM models, leading to so called ‘target independence’ [119]. Compensatory activation of alternative RTKs can also be achieved through epigenetic mechanisms. For instance, PDGFRB expression is induced by EGFR inhibitors, leading to EGFRi resistance [120]. Alternative reactivation of downstream signaling pathways, such as PI3K signaling by PTEN loss, is another resistance mechanism found with EGFR TKis [121]. There is also sufficient preclinical evidence showing that activation of MET, IGF1R, ERBB2, or ERBB3 confers resistance to EGFR TKis [122,123].

Antitumor efficacy to PDGFR-targeting drugs seems to depend on similar compensatory signaling mechanisms, as the co-expression of ERBB3, IGF1R and TGFBR2 in PDGFR expressing glioblastoma cells contributes to PDGFR inhibitor resistance [124]. In addition, insulin can promote resistance to PDGFR inhibition in gliomas driven by PDGFB [125]. Thus, intriguingly the individual mechanisms to escape the inhibitory effects of TKIs share common signaling components, which can be informative for the design of more effective combination therapies.

### 4.5. A Role for Corticoids in the Design of RTKi Trials?

Neuro-inflammation and peritumoral edema is one of the main factors affecting the wellbeing of GBM patients with regard to neurologic symptoms, such as blurred vision, dizziness, nausea, aphasia, and headaches [126]. These comorbidities have great impact in the course of the disease. Glioblastoma patients are very often administered glucocorticoids (mainly dexamethasone) to manage brain swelling, and increasing evidence is mounting suggesting a negative correlation between dexamethasone administration and overall survival [106,127,128,129]. As has been described in many cell and tumor types, glucocorticoid receptor activation leads to wide transcriptomic rewiring. Importantly, in GBM dexamethasone promotes the hyperactivation of the PDGFR pathway, establishing a transcriptional program that promotes radio-resistance through bypassing the mitotic checkpoint via modulation of the Spindle Assembly Complex [106]. However, treatment with multikinase inhibitors targeting PDGFR overcame the radioprotective activity of dexamethasone. Moreover combining dexamethasone with TKIs produced a synergistic inhibitory effect on tumor cell growth in vitro and in vivo [106]. These findings suggest a complex interplay between dexamethasone and TKIs. However, glucocorticoid administration has not been taken into account when designing, developing, and analyzing the data of clinical trials assessing TKIs.

## 5. Conclusions

Despite global efforts to incorporate targeted therapy into GBM management, the Stupp regime continues to be the standard of care for GBM patients. Inhibitors of RTKs that drive GBM progression have so far failed to produce significant clinical results. In the face of the dire prognosis that GBM patients endure, this situation is clearly discouraging. However, considering the weaknesses in the design of previous trials, it appears there is room for improvement, and TKIs should not yet be discarded as potentially therapeutic opportunities in GBM. In this review, we have aimed to highlight the factors that might need to be taken into account in order to better conceive future clinical trials testing TKIs in GBM patients. There is compelling evidence to include molecular testing for TKI target expression in order to enable patient stratification. Moreover, such information will allow for the meaningful analysis of patient data from a completed trial. Notably, we propose that analysis of protein expression levels, together with gene amplification/mutation, should be implemented to provide a more accurate view of the molecular landscape for each patient. For instance, immunohistochemical analyses could inform about the impact of tumor heterogeneity on patients’ response to treatment. Since both genetic and protein expression analysis can be easily performed, clinicians have the opportunity to use either of them as inclusion criteria, or for posterior correlation analyses between clinical response and molecular profile. Given the increased evidence demonstrating that the mutational and/or molecular landscape of recurrent tumors can differ from that of newly diagnosed tumor tissue, it would be important to, at least, obtain biopsies of the recurrent tumor for analyses if resection is not an option. Alternatively, the option of assessing TKIs in newly diagnosed GBM patients would be the most informative option. In this regard, the combination of present adjuvant therapies (radiotherapy and/or temozolomide) together with TKIs appears to be an understudied therapeutic opportunity.

## Figures and Tables

**Table 1:** Receptor Tyrosine Kinase inhibitors targeting drivers of GBM trialed in glioblastoma patients.

Drug	Trade Name	Human Targets	First FDA Approval		Clinical Use in Cancer
IMATINIB	GLEEVEC	KIT, ABL1, PDGFRB	2001	CML	CML, GIST
GEFITINIB	IRESSA	EGFR	2003	NSCLC	NSCLC
ERLOTINIB	TARCEVA	EGFR	2004	NSCLC	NSCLC, PCa
SORAFENIB	NEXAVAR	BRAF, PDGFRB, FLT1, FLT4, KDR, FLT3, RAF1, RET, KIT	2005	RCC	RCC
SUNITINIB	SUTENT	PDGFRB, PDGFRA, KIT, FLT3, CSF1R, FLT1, FLT4, KDR	2006	RCC	RCC
DASATINIB	SPRYCEL	FYN, SRC, LCK, YES1, BLK, HCK, LYN, FRK, FGR, SRMS, EPHA2, PDGFRB, ABL1, KIT	2006	CML	CML
LAPATINIB	TYVERB	EGFR, ERBB2	2007	BCa	BCa
PAZOPANIB	VOTRIENT	PDGFRB, PDGFRA, FGFR3, FGFR1, FLT1, FLT4, KDR, ITK, CSF1R, KIT, LCK	2009	RCC	RCC, SARCOMA
VANDETANIB	CAPRELSA, ZACTIMA	ERBB2,3,4, EGFR, FLT1, FLT4, KDR, PTK6, EPHA (1–7,10) PHA8, EPHB (1–4,6) RET, SRC,TEK	2011	TC	TC
REGORAFENIB	STIVARGA	TEK, RAF1, FGFR1, DDR2, BRAF, RET, MAPK11, FLT1,4, KDR, FGFR2, FRK, PDGFRB, PDGFRA, ABL1, KIT	2012	CRC, GIST	CRC, GIST, HCC
CABOZANTINIB	CABOMETYX, COMETRIQ	KDR, MET	2012	TC	TC
AXITINIB	INLYTA	FLT1, FLT4, KDR	2012	RCC	RCC, SARCOMA
PONATINIB	ICLUSIG	BCR, ABL1, PDGFR, FGFR, EPHR, KIT, SRC RET, FLT3	2012	CML	CML, Ph+ALL
AFATINIB	GIOTRIF	ERBB2, ERBB4, EGFR	2013	NSCLC	NSCLC
NINTEDANIB	OFEV, VARGATEF	FLT1, FLT4, KDR, PDGFRB, PDGFRA, FGFR3, FGFR1, FGFR4, FGFR2	2014	PF	NSCLC
OSIMERTINIB	TAGRISSO	EGFR	2015	NSCLC	NSCLC
NERATINIB		EGFR, ERBB4, ERBB2	2017	BCa	BCa
DACOMITINIB		ERBB2, EGFR, ERBB4	2018	NSCLC	NSCLC
INFIGRATINIB		FGFR3, FGFR1, FGFR4, FGFR2	2021	CCA	CCA

NSCLC: non-small cell lung cancer; TC: thyroid cancer, CRC: Colorectal Cancer; GIST: gastrointestinal stromal tumor; HCC: hepatocellular carcinoma; RCC: renal cell carcinoma; CML: chronic myeloid leukemia; Ph+ALL: Philadelphia chromosome-positive acute lymphoblastic leukemia; PF: pulmonary fibrosis BCa: breast cancer; CCA: metastatic cholangiocarcinoma.

**Table 2:** Clinical trials of Receptor Tyrosine Kinase Inhibitors in Newly diagnosed Glioblastoma.

Drug	NTC Number	Phase	Status	Start Date
GEFITINIB	NCT00238797	2	Completed	1 February 2003
VATALANIB	NCT00385853	1	Completed	1 September 2006
SORAFENIB	NCT00544817	2	Completed	1 April 2007
DASATINIB	NCT00895960	2	Withdrawn	1 May 2009
SUNITINIB	NCT01100177	2	Completed	1 June 2009
TANDUTINIB	NCT00904852	1	Withdrawn	1 June 2009
AFATINIB	NCT00977431	1	Completed	17 September 2009
AXITINIB	NCT01508117	2	Terminated	1 August 2011
SUNITINIB	NCT02928575	2	Unknown status	1 August 2012
LAPATINIB	NCT01591577	2	Active, not recruiting	7 December 2012
PAZOPANIB	NCT02331498	1	Active, not recruiting	1 June 2015
NERATINIB	NCT02977780	2	Recruiting	9 February 2017
EPITINIB	NCT03231501	1	Recruiting	26 January 2018
ANLOTINIB	NCT04119674	1	Recruiting	15 January 2019
REGORAFENIB	NCT03970447	2	Recruiting	30 July 2019
ANLOTINIB	NCT04157478	2	Not yet recruiting	1 January 2020
ANLOTINIB	NCT04725214	2	Recruiting	15 January 2021

**Table 3:** Clinical trials of receptor tyrosine kinase inhibitors in recurrent or progressive glioblastoma.

Drug	NTC Number	Phase	Status	Start date
ERLOTINIB	NCT00337883	2	Completed	1 July 2003
IMATINIB	NCT00171938	2	Terminated	1 April 2004
IMATINIB	NCT00154375	3	Completed	1 October 2004
GEFITINIB	NCT00250887	2	Completed	1 July 2005
IMATINIB	NCT00290771	2	Terminated	1 February 2006
ERLOTINIB	NCT00301418	1	Completed	1 March 2006
SUNITINIB	NCT00606008	2	Completed	1 March 2007
SUNITINIB	NCT00864864	0	Completed	1 May 2007
CEDIRANIB	NCT00503204	1	Completed	1 September 2007
SORAFENIB	NCT00597493	2	Completed	1 September 2007
SUNITINIB	NCT00535379	2	Unknown status	1 October 2007
CABOZANTINIB	NCT00704288	2	Completed	1 May 2008
SUNITINIB	NCT00923117	2	Terminated	1 June 2008
CEDIRANIB	NCT00777153	3	Completed	1 October 2008
VANDETANIB	NCT00821080	1	Completed	1 October 2008
DASATINIB	NCT00892177	2	Completed	1 October 2009
DASATINIB	NCT00948389	1	Terminated	1 October 2009
DACOMITINIB	NCT01112527	2	Completed	1 April 2010
NINTEDANIB	NCT01251484	2	Completed	1 January 2011
CEDIRANIB	NCT01310855	2	Terminated	1 May 2011
GEFITINIB	NCT01310855	2	Terminated	1 May 2011
ERLOTINIB	NCT01110876	1	Terminated	1 June 2011
DACOMITINIB	NCT01520870	2	Completed	1 February 2012
NINTEDANIB	NCT01666600	1	Terminated	1 August 2012
SORAFENIB	NCT01817751	2	Active, not recruiting	11 April 2013
DOVITINIB	NCT01972750	1	Unknown status	1 October 2013
INFIGRATINIB	NCT01975701	2	Completed	9 December 2013
REGORAFENIB	NCT02926222	2	Active, not recruiting	1 November 2015
CRENOLANIB	NCT02626364	2	Completed	1 April 2016
SORAFENIB	NCT01434602	1	Active, not recruiting	11 April 2016
TESEVATINIB	NCT02844439	2	Completed	1 June 2016
AXITINIB	NCT03291314	2	Completed	3 May 2017
CEDIRANIB	NCT02974621	2	Active, not recruiting	15 September 2017
SUNITINIB	NCT03025893	2	Recruiting	31 August 2018
OSIMERTINIB	NCT03732352	2	Active, not recruiting	28 November 2018
REGORAFENIB	NCT03970447	2	Recruiting	30 July 2019
REGORAFENIB	NCT04051606	2	Recruiting	31 July 2019
INFIGRATINIB	NCT04424966	0	Recruiting	21 July 2020
ANLOTINIB	NCT04547855	2	Recruiting	11 September 2020

**Table 4:** Inclusion criteria (%) in GBM TKIs clinical trials.

Clinical Trials	Inclusion Criteria	Newly Diagnosed	Recurrent	Total
All	Others	30.91%	69.09%	100%
	TKR expression or mutation	5.88%	18.42%	14.54%
Currently ongoing	Others	47.06%	52.94%	100%
	TKR expression or mutation	12.50%	33.33%	23.53%
